# A systematic review of economic evaluations of conservative treatments for chronic lower extremity musculoskeletal complaints

**DOI:** 10.1093/rap/rky030

**Published:** 2018-09-10

**Authors:** Linda Fenocchi, Jody L Riskowski, Helen Mason, Gordon J Hendry

**Affiliations:** 1 Musculoskeletal Health Research Group, School of Health and Life Sciences, Glasgow Caledonian University; 2 Yunus Centre for Social Business & Health, Glasgow Caledonian University, Glasgow, UK

**Keywords:** systematic review, economic evaluation, lower extremity musculoskeletal conditions, cost effectiveness, conservative interventions

## Abstract

**Objective:**

The aim was to appraise and synthesize studies evaluating the clinical and cost effectiveness of conservative interventions for chronic lower extremity musculoskeletal (MSK) conditions and describe their characteristics, including the type of economic evaluation, primary outcomes and which conditions.

**Methods:**

The search strategy related to economic evaluations of lower limb MSK conditions that used conservative therapies. Eight electronic databases were searched (CENTRAL, MEDLINE, PubMed, EMBASE, CINAHL, PEDro, NHSEED and Proquest), as were the reference lists from included articles. The quality of articles was appraised using a modified version of the economic evaluations’ reporting checklist (economic) and The Cochrane Collaboration’s tool for assessing risk of bias (clinical).

**Results:**

Twenty-six studies were eligible and included in the review. Economic evaluations of conservative interventions for OA or pain affecting the knee/hip (*n* = 25; 93%) were most common. The main approaches adopted were cost–utility analysis (*n* = 17; 68%) or cost–effectiveness analysis (*n* = 5; 19%). Two studies involved interventions including footwear/foot orthoses; for heel pain (*n* = 1; 4%) and overuse injuries (*n* = 1; 4%). Fifty per cent of economic evaluations adopted the EQ-5D-3L as the primary outcome measure for quality of life and quality-adjusted life year calculations.

**Conclusion:**

Economic evaluations have been conducted largely for exercise-based interventions for MSK conditions of the hip and knee. Few economic evaluations have been conducted for other clinically important lower limb MSK conditions. A matrix presentation of costs mapped with outcomes indicated increasing costs with either no difference or improvements in clinical effectiveness. The majority of economic evaluations were of good reporting quality, as were the accompanying clinical studies.

**Key messages**

- This is a comprehensive systematic review of economic evaluations of conservative treatments for common lower extremity musculoskeletal conditions.
- Economic evaluations of hip and knee OA dominate cost-effectiveness literature for lower extremity musculoskeletal conditions.
- The reporting quality of clinical and economic evidence for lower extremity musculoskeletal conditions is generally good.

## Introduction

Worldwide, >20% of the population have a musculoskeletal (MSK) condition [1]. These conditions are one of the main drivers of increasing years lived with disability [2, 3], and their management has major implications for health-care resource use. A wide range of inflammatory and degenerative conditions are classed as MSK conditions [4], and they are often characterized by pain, limitations on physical function and reductions in health-related quality of life [5]. For many MSK conditions, the first line of management is conservative treatment. This may include options such as exercise programmes, self-management education and physical therapies [6]. However, evidence of clinical and cost effectiveness for conservative interventions for MSK conditions remains equivocal. Although there is a growing evidence base for clinical effectiveness for some conservative treatments, the evidence for cost effectiveness is often lacking. This is problematic given that health-care systems must deal with resource allocation constraints. To maximize health using the resources available, it is necessary to make choices between competing claims.

The overall aim of economic evaluations in health care is to aid decision-makers to make efficient and equitable decisions [7]. Economic evaluation involves the comparison of two or more health-care interventions, typically comparing a new intervention with usual care, in terms of the costs and the consequences [7, 8]. The inclusion of the outcomes in addition to costs is crucial if we are to determine which interventions produce the greatest health gain for our given budget. (For a glossary of economic terms, see [9].)

Systematic reviews are useful to assess evidence of effects, adverse effects and health-related quality of life and to identify gaps in research [10]. Systematic reviews of economic evaluations can be used to establish the current state of the art in economic evaluations of interventions that assess cost effectiveness and provide a foundation for higher methodological standards [8, 11]. Previous reviews of the cost effectiveness of non-pharmacological and non-surgical treatments for MSK conditions have focused on specific patients or interventions [12, 13], whereas in the present study we sought to increase the scope to include any attempt to compare costs with benefits for any lower extremity MSK condition.

Accordingly, the aims of this review were to identify and critically appraise the current evidence of clinical and cost effectiveness of conservative interventions for the treatment of lower extremity MSK conditions to determine whether there is sufficient evidence to inform policy and practice [14] and to identify and describe the characteristics of these economic evaluations, including the type of economic evaluation, primary outcomes, which lower extremity MSK conditions, and a synthesis of their results.

## Methods

### Protocol

The protocol for the systematic review was submitted and approved *a priori* (PROSPERO 2015: CRD42015024441 [15]) and followed the preferred reporting items for systematic reviews and meta-analyses (PRISMA) guidelines [16].

### Search strategy

Peer-reviewed literature was searched according to a predefined strategy using a combination of MeSH related to MSK and physical body location and key words (any field), including text words related to economic evaluation (Supplementary Table S1, available at *Rheumatology* online). The strategy was wide in scope in order to be inclusive so that relevant studies were returned.

The search was conducted for studies published up to 10 September 2017. Eight databases were searched: Cochrane Central Register of Controlled Trials (CENTRAL), Medical Literature Analysis and Retrieval System Online (MEDLINE), PubMed, Excerpta Medica database (EMBASE), Cumulative Index to Nursing and Allied Health Literature (CINAHL), Physiotherapy Evidence Database (PEDro), NHS Economic Evaluation Database (NHS EED; addition of bibliographic records to NHS EED ceased after 31 March 2015) and Proquest. Results were imported to Endnote (v.7.1; Thomson Reuters).

### Inclusion criteria

Articles reporting an economic evaluation of health professional-delivered conservative intervention for the treatment of MSK conditions of the lower extremities were the focus of the systematic review (Table 1). Medical treatments such as pharmacological, homeopathic and surgical interventions were excluded. Studies that were primarily clinical but had some analysis of cost in relationship to benefit (using an economic tool or method to calculate outcome) were included before a process of screening to ascertain whether they met economic evaluation definitions (UK classification system [7]). Articles reporting embedded economic evaluations, including randomized controlled trials or quasi-randomized controlled trails, controlled trials and pilot studies, were eligible for inclusion.

**Table 1:** Systematic review study criteria

Criteria	Description
Study design	Included studies were economic evaluation articles with their associated clinical article or studies reporting embedded economic evaluations of conservative, non-pharmacological and non-surgical interventions for lower extremity MSK conditions. Excluded studies reported surgical or pharmacological interventions for upper extremity MSK conditions.
Study participants	Adult humans (as defined by study). Included: lower extremity [hip, thigh, knee, calf, ankle, foot and toes(s)] MSK conditions that originate in, and having a mechanical aetiology, affect the MSK system. Excluded: systemic conditions (such as cancer, vascular, multiple sclerosis, gout, diabetes)
Study time frame	No restrictions
Outcomes measures	Studies were assessed for: Scope and range of evidence of clinical effectiveness and cost effectiveness. Quality of the evidence. Identification of common outcome measures used, clinical and/or economic.
Analysis	Descriptive synthesis, summary of findings table, decision matrix linking clinical effectiveness with cost.

MSK: musculoskeletal.

Adult lower extremity MSK conditions considered theoretically to have a mechanical aetiology (such as OA, stress trauma, overuse injuries or biomechanical misalignment) were included. In addition, only conditions affecting the lower limb (International Classification of Functioning, Disability and Health [17] structures of the lower extremity, s750; hip, s75001; thigh, s7500; knee, s75011; ankle and foot, s7502) were considered.

### Exclusion criteria

Pharmacological, homeopathic or surgical interventions were excluded. Systemic conditions, such as diabetes or RA, and neurological conditions whereby the primary condition was not MSK in origin, were excluded. Lower extremity MSK conditions resulting from acute or injury trauma (e.g. athletic ankle sprain, professional ballet injuries) were excluded. Musculoskeletal complaints in the axial regions, torso and upper extremity were excluded. Non-peer-reviewed documentation, such as commentaries, letters and editorials, were excluded. Articles were limited to those available in English. No restrictions were placed on publication date.

### Study selection

Studies were identified, selected and appraised using methodology in line with The Cochrane Handbook for Systematic Reviews [10]. Title screening of studies was undertaken by one reviewer (L.F.), using the key words and MeSH terms to determine whether the title warranted further consideration for review. This was followed by independent review of abstracts, then full text, by two authors (L.F. and G.J.H.). At each stage, reviewer agreement or disagreement was recorded, with justification. For included articles, if the economic evaluation referred to a primary clinical paper then a copy of that paper was sought and included in the review. Economic and accompanying clinical articles were treated as one study. Reference lists of included studies were hand searched.

### Data extraction

The data-extraction tool for this review included patient population, study design, economic evaluation method, intervention, follow-up and clinical and cost-effectiveness outcomes. This data-extraction tool was used independently by two authors (L.F. and G.J.H.).

### Quality assessment

The reporting quality of economic evaluations of the included studies was assessed independently by two authors (L.F. and G.J.H.) using a modified version of the economic evaluations’ reporting checklist [18, 19]. The modified checklist included 13 items (plus an additional two items applicable for decision analytical modelling studies). The included items were selected based on their direct relevance to economic evaluations of single clinical treatment studies and the specific research question for the systematic review. Clinical studies were evaluated for their quality separately using The Cochrane Collaboration’s tool for assessing risk of bias [10, 20].

### Synthesis of evidence

Evidence of cost effectiveness relative to clinical effectiveness was summarized using a matrix [8]. The matrix was developed to aid discussion about the choices between health-care interventions that are available to managers and clinicians. It provides a visual representation and summary of available clinical and economic evidence. By mapping these two sources of evidence together, it demonstrates both technical efficiency (which interventions are offering most clinical benefit for the resources used) and opportunity cost considerations (what the next best option would have offered) at the same time. Statements of clinical effectiveness and evidence of cost effectiveness were accepted as reported by study authors. This was a pragmatic decision based on the fact that all included studies had been peer reviewed. Clinical effectiveness relative to the treatment comparator is mapped horizontally. Evidence of impact on resources in terms of marginal change is mapped vertically. The main feature of utility of the matrix is that it provides easily accessible information to aid decision-making by health-care providers concerning treatment options.

It is recommended that only studies appraised as good quality are mapped in the matrix [8]. Studies were included in the matrix if they had a quality score between 70 and 100% for both clinical reporting [10, 20] and economic reporting [18, 19]. Reported conclusions about clinical and cost effectiveness were mapped to one another. For studies that involved more than one intervention–comparator pairing, these were mapped by each individual intervention to the comparator (13 studies, 21 pairs). Studies with insufficient information about intervention–comparator pairings could not be mapped (13 studies).

## Results

### Search results

**Fig. 1:**
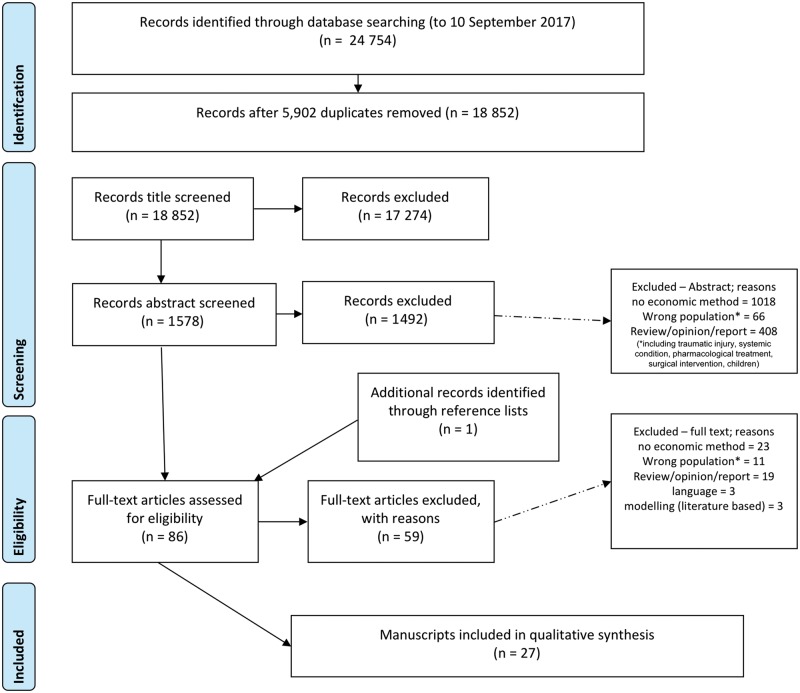
PRISMA diagram for systematic review PRISMA: preferred reporting items for systematic reviews and meta-analyses.

A total of 24 754 records were returned as a result of searching, and after removing duplicates there were 18 852 records (Fig. 1). Based on the inclusion and exclusion criteria, the review of titles excluded 17 274 records, leaving 1578. At the abstract stage, 1492 were excluded, leaving 86 records for full text review. Twenty-seven articles met the inclusion criteria, including one additional article identified through reference lists. Of these, two economic papers [21, 22] reported on the same analysis of the same study, meaning that in total there were 27 articles representing 26 unique studies.

### Studies included in the review

Data extracted from 26 included studies were from the economic articles [21–47] and their associated clinical papers [48–64] (Table 2). The majority of studies were written as separate economic evaluations (*n* = 15; 58%) with an associated clinical paper, whereas a minority included embedded reporting of economic evaluation (*n* = 11; 42%) in the parent article. Using the UK definition of economic evaluation approaches [7], there were: 17 cost–utility analyses (CUA), five cost–effectiveness analyses (CEA), three cost–consequence analyses (CCA), and one cost-minimization analysis (CMA). Articles were published between 1999 and 2017.

**Table 2:** Studies included in the review

Citation	MSK condition	Economic evaluation approach (UK definitions)	Intervention (number of participants)	Comparator (number of participants)	Clinical tool (primary outcome)	Clinical tool change	Economic outcome tool-quality of life	Health-related quality of life (economic) tool change
Barton *et al.* (2009) [23] (clinical [50])	Knee pain	CUA	Dietary intervention plus strengthening exercises (*n*=109)	Leaflet provision (equivalent to standard care) (*n*=76)	WOMAC	⇧	EQ-5D-3L	⇧
			Dietary intervention (*n*=122)	Leaflet provision (equivalent to standard care) (*n*=76)	WOMAC	⇔	EQ-5D-3L	⇧
			Strengthening exercises (*n*=82)	Leaflet provision (equivalent to standard care) (*n*=76)	WOMAC	⇔	EQ-5D-3L	⇔
Bennell *et al.* (2016) [24] (clinical not yet available)	Knee OA	CUA	PCST and exercise (*n*=73)	Exercise (*n*=75)	VAS knee pain plus WOMAC	⇧	AQoL-6D	⇧
			PCST and exercise (*n*=73)	PCST (*n*=74)	VAS knee pain plus WOMAC	⇧	AQoL-6D	⇧
			PCST (*n*=74)	Exercise (*n* =75)	VAS knee pain plus WOMAC	⇔	AQoL-6D	⇔
Ciani *et al.* (2017) [46] (clinical [60])	Knee OA	CUA	Mud-bath therapy (*n*=53)	Usual care (*n*=50)	WOMAC	⇧	EQ-5D-3L	⇧
Cochrane *et al.* (2005) [25]	Hip OA + knee OA	CUA	Water-based exercise (*n*=153)	Usual care (*n*=159)	WOMAC	⇧	SF36, EQ-5D-3L	
Coupé *et al.* (2007) [26] (clinical [57])	Hip OA + knee OA	CUA	Behavioural graded activity (*n*=56)	Usual care (*n*=66)	VAS knee pain plus WOMAC	⇔	EQ-5D-3L	⇔
Hurley *et al.* (2007) [28, 53] (clinical [49])	Knee pain	CUA	Exercise-based rehabilitation programme (*n*=278)	Usual care (*n*=140)	WOMAC	⇧	EQ-5D-3L	⇔
			Individual exercise-based rehabilitation programme (*n*=146)	Usual care (*n*=140)	WOMAC	⇧	EQ-5D-3L	⇔
			Group exercise-based rehabilitation programme (*n*=132)	Usual care (*n*=140)	WOMAC	⇧	EQ-5D-3L	⇔
			Group exercise-based rehabilitation programme (*n*=132)	Individual exercise-based rehabilitation programme (*n*=146)	WOMAC	⇔	EQ-5D-3L	⇔
Hurley *et al.* (2012) [27]	Knee pain	CUA	Exercise-based rehabilitation programme (*n*=189)	Usual care (*n*=94)	WOMAC	⇧	As clinical	–
Jessep *et al.* (2009) [29]	Knee OA	CEA	Exercise-based rehabilitation programme (*n*=29)	Outpatient physiotherapy (*n*=35)	WOMAC	⇔	EQ-5D-3L	⇔
Juhakoski *et al.* (2011) [30]	Hip OA	CCA	Combined exercise and usual care (*n*=60)	Usual care (*n*=58)	WOMAC	⇔	RAND-36 (SF-36)	⇔
Lord *et al.* (1999) [31] (clinical [46])	Knee OA	CMA	Nurse-led education (*n*=105)	Usual care (*n*=65)	WOMAC	⇔	SF-36	⇔
Losina *et al.* (2015) [32] (clinical [51, 52])	Knee OA	CUA	Arthroscopic partial meniscectomy (*n*=351)	Physical therapy (*n*=164)	WOMAC	⇔	EQ-5D-3L	–
Marra *et al.* (2014) [33] (clinical [57])	Knee OA	CUA	Pharmacist-led health care (*n*=66)	Usual care (*n*=73)	Arthritis Foundation quality indicators for the management of OA	⇧	HUI3	⇔
Mazzuca *et al.* (1999) [34] (clinical [54])	Knee OA	CCA	Education (individualized arthritis self-care instruction) (*n*=105)	Attention control (*n*=106)	HAQ	⇔	None (health-care utilization and costs data)	–
McCarthy *et al.* (2004) [21]	Knee OA	CUA	Class-based exercise programme + home exercise programme (*n*=111)	Home exercise programme (*n*=103)	Timed measure of three locomotor activities	⇔	EQ-5D-3L	⇔
Patel *et al.* (2009) [35] (clinical [45])	Hip OA + knee OA	CUA	Arthritis self-management programme plus an education booklet (*n*=406)	Education booklet (reflects standard care) (*n*=406)	SF-36	⇔	EQ-5D-3L	⇔
Pinto *et al.* (2013) [36] (clinical [44])	Hip OA + knee OA	CUA	Manual therapy (*n*=54)	Usual care (*n*=51)	WOMAC	⇧	SF12v2 (SF-6D)	⇧
			Exercise therapy (*n*=51)	Usual care (*n*=51)	WOMAC	⇧	SF12v2 (SF-6D)	⇧
			Manual and exercise therapy (*n*=50)	Usual care (*n*=51)	WOMAC	⇧	SF12v2 (SF-6D)	⇧
Reinhold *et al.* (2008) [37] (clinical [58])	OA	CUA	Acupuncture (*n*=246)	Delayed acupuncture (equivalent to no treatment) (*n*=243)	WOMAC	⇧	SF-36 (SF-6D)	⇧
Richardson *et al.* (2006) [22]	Knee OA	–	–	–	–	–	–	–
Rome *et al.* (2004) [38]	Heel pain	CUA	Accomodative orthoses (*n*=22)	Functional orthoses (*n*=26)	FHSQ	⇧	EQ-5D-3L	⇧
Sevick *et al.* (2000) [39] (clinical [47])	Knee OA	CEA	Aerobic exercise (*n*=144)	Education booklet (reflects standard care) (*n*=149)	Investigator-developed questionnaire	⇧	Investigator-developed questionnaire	–
			Resistance exercise (*n*=146)	Education booklet (reflects standard care) (*n*=149)	Investigator-developed questionnaire	⇧	Investigator-developed questionnaire	–
			Resistance exercise (*n*=146)	Aerobic exercise (*n*=144)	Investigator-developed questionnaire	⇧	Investigator-developed questionnaire	–
Sevick *et al.* (2009) [47] (clinical [55])	Knee OA	CEA	Diet (*n*=82)	Healthy lifestyle control (attention control comparison) (*n*=78)	WOMAC	⇧	As clinical	–
			Exercise (*n*=80)	Healthy lifestyle control (attention control comparison) (*n*=78)	WOMAC	⇔	As clinical	–
			Diet + exercise (*n*=76)	Healthy lifestyle control (attention control comparison) (*n*=78)	WOMAC	⇧	As clinical	–
			Diet + exercise (*n*=76)	Diet (*n*=82)	WOMAC	⇧	As clinical	–
			Diet + exercise (*n*=76)	Exercise (*n*=80)	WOMAC	⇧	As clinical	–
			Exercise (*n*=80)	Diet (*n*=82)	WOMAC	⇔	As clinical	–
Stan *et al.* (2015) [40]	Knee OA	CUA	Unilateral TKA (non-operated knee) (*n*=30)	Rehabilitation care (*n*=30)	EQ-5D-3L	⇧	As clinical	–
			TKA following HTO (*n*=30)	Rehabilitation care (*n*=30)	EQ-5D-3L	⇧	As clinical	–
			Unilateral TKA (non-operated knee) (*n*=30)	TKA following HTO (*n*=30)	EQ-5D-3L	⇔	As clinical	–
Tan *et al.* (2016) [41]	Hip OA	CUA	Exercise therapy added to GP care (*n*=101)	GP care (*n*=102)	HOOS	[Forthcoming paper]	EQ-5D-3L	⇔
Thomas *et al.* (2005) [42] (clinical [56])	Knee pain	CEA	Exercise + telephone support + placebo (*n*=114)	Exercise + telephone support (*n*=121)	WOMAC	⇔	As clinical	–
			Placebo (*n*=78)	No intervention (*n*=78)	WOMAC	⇔	As clinical	–
			Exercise therapy (*n*=235)	Combined no intervention and placebo (*n*=156)	WOMAC	⇧	As clinical	–
			Monthly telephone support (*n*=160)	Combined no intervention and placebo (*n*=156)	WOMAC	⇔	As clinical	–
			Exercise + telephone support (combining exercise + telephone support with exercise + telephone support + placebo) (*n*=235)	Combined no intervention and placebo (*n*=156)	WOMAC	⇧	As clinical	–
Torkki *et al.* (2002) [43]	Overuse injuries	CCA	New, individually adjusted footwear with good shock-absorbing properties (*n*=86)	Subjects’ own, used footwear (*n*=90)	Investigator-developed questionnaire	⇔	As clinical	–
Whitehurst *et al.* (2011) [44] (clinical [48])	knee OA	CUA	Advice and exercise plus true acupuncture (*n*=117)	Advice and exercise (*n*=116)	WOMAC	⇧	EQ-5D-3L	⇔
			Advice and exercise plus true acupuncture (*n*=117)	Advice and exercise plus non-penetrating acupuncture (*n*=119)	WOMAC	⇔	EQ-5D-3L	⇔
Witt *et al.* (2006) [45, 62] (clinical [58, 59])	Chronic pain	CUA	Acupuncture (*n*=322)	Usual care (*n*=210)	WOMAC	⇧	SF-36	⇧

⇧: statistically significant change; ⇔: not a statistically significant change. Statistical significance is based on the author’s definition. AQoL-6D: assessment of quality of life – 6D scale; EQ5D: EuroQol 5 dimensions; EQ-VAS: EuroQol visual analog scale; FHSQ: foot health status questionnaire; GP: general practitioner; HOOS: hip disability and osteoarthritis outcome score; HUI3: health utilities index mark 3; RAND-36: Finnish-validated SF-36-item health survey; SF-6D: short form 6 dimensions; SF-12: short form 12; SF-36: Short form 36; VAS: visual analog scale; PCST: Pain Coping Skills Training; TKA: total knee arthroplasty.

### Conservative interventions

The conservative interventions of the included economic evaluations were of exercise-based intervention (*n* = 11; 42%), education (*n* = 3; 12%), combined exercise and education (*n* = 3; 12%), combined exercise and diet (*n* = 2; 8%), acupuncture (*n* = 3; 12%), footwear/orthoses (*n* = 2; 8%), physical therapy (*n* = 1; 4%) and mud-bath therapy (*n* = 1; 4%). Studies were largely focused on interventions involving an exercise component for OA or pain management affecting the knee/hip (*n* = 24; 92%). The remaining studies evaluated conservative interventions for heel pain (*n* = 1; 4%) and lower limb overuse injuries (*n* = 1; 4%).

### Primary outcome measures

Of the included studies, the most commonly used outcomes measures adopted for evaluating the clinical and cost effectiveness of conservative treatments were the WOMAC (CEA) and the generic preference-based measure EQ-5D-3L, developed by the EuroQoL group (CUA). Two studies were concerned with foot and ankle conditions, using the foot health status questionnaire [38] and an investigator-developed questionnaire [43], respectively.

Five generic preference-based outcome measures were used by 21 economic evaluations: EQ-5D-3L (12 CUAs, 1 CEA and 1 CCA), SF-36 [2 CUAs, 1 CEA (RAND-36) and 1 CMA], SF-12v2 [1 CUA (SF-6D)], AQoL-6D (1 CUA) and HUI-3 (1 CUA). Each of these tools produces utility values that can be used in the calculation of quality-adjusted life years, essential for comparisons across different diseases. Three studies collected only clinical measures of health for hip OA and knee OA and were therefore restricted to CEA methodology (i.e. cost per unit of improvement in condition-specific outcome measures) [27, 42, 47]. One study used an investigator-developed questionnaire to undertake a CEA [39]. Two studies collected utilization of health care and cost data and conducted a CCA [34, 43].

### Quality of the evidence

The reporting quality of economic evaluations and related clinical studies was generally good (for this review, defined as scoring between 70 and 100% for items on each reporting quality checklist; Table 3). Ten studies reported on all 13 of the economic evaluations’ reporting list items that were selected for appraisal [21, 22, 24, 27, 28, 31, 33, 35, 36, 41, 44]. A further nine studies reported on >70% of the items [23, 25, 26, 32, 37, 38, 42, 46, 47], and four studies were considered to have reported on at least half of the key elements [29, 34, 39, 43]. The remaining three were appraised to have poor reporting quality [30, 40, 45]. Witt *et al.* [45] did not report adequately on resource use and methods for estimation of quantities and unit costs. For Juhakoski *et al.* [30], reporting of methods for estimation and quantities of costs was restricted because they were using study data collected for clinical effectiveness considerations, not economic. The paper by Stan *et al.* [40] was judged to have poor reporting quality for both clinical and economic considerations. Sampling strategy was not reported, nor why EQ-5D-3L administration was at different follow-up intervals for different intervention arms.

**Table 3:** Summary of interventions by lower extremity (26 included studies)

Anatomical location of MSK condition						
Intervention (examples)	Lower limbs (general)	Hip and knee	Hip	Knee	Foot	Total
Acupuncture (deep needling, superficial needling, true acupuncture, non-penetrating)		2		1		3
Education (education booklet, self-care education, nurse-led education programme)		1		2		3
Exercise (aerobic exercise, resistance exercise, exercise aimed at increasing lower limb strength and endurance and improving balance)		2	2	7		11
Exercise + diet (healthy eating diet + quadriceps strengthening exercises)				2		2
Exercise + education (behavioural graded activity integrating the concepts of operant conditioning with exercise therapy, supervised exercise and pain-management and coping strategies)		1		2		3
Footwear (functional orthoses, accommodative orthoses, sports shoe)	1				1	2
Mud-bath therapy (mud-packs and hot mineral baths in addition to usual treatment)				1		1
Physical therapy (manual physiotherapy)				1		1
Total	1	6	2	16	1	

Sixteen clinical studies were appraised as good quality with low risk of bias [21, 24, 25, 27, 30, 43, 46, 48, 49, 51–53, 55, 59–61], six appraised as medium risk of bias [29, 38, 41, 57, 58, 62], and four as high risk of bias [37, 40, 50, 54]. Blinding and attrition risks were common to most of the studies. As would be expected with interventions involving such treatments as exercise or footwear, it was not possible to blind participants and assessors. Higher risk of bias owing to incomplete outcome data was also noted in more than half of the studies [21–23, 25, 29–31, 33, 37–40, 42, 44, 45, 50–52, 54, 60], although this is not unusual for interventions that require adherence, such as exercise therapies. It should be noted that Tan [41] reported that a clinical article is forthcoming; therefore, the judgement about risk of bias was made on the basis of the available evidence in the economic evaluation article.

### Cost effectiveness of interventions

Economic evidence, for studies with a quality score between 70 and 100% for both clinical reporting [10, 20] and economic reporting [18, 19] (Table 4), was synthesized in a matrix (Table 5). The reported evidence for exercise interventions for hip/knee OA is mixed, with studies reporting in A1 (evidence of greater clinical effectiveness and reductions in costs) [25, 28, 42, 46], B1 (evidence of greater clinical effectiveness with no difference in costs) [24, 28] and B2 (evidence of no difference in clinical effectiveness and no difference in costs reported, relative to comparator) [21, 22, 26, 44], C1 (evidence of greater clinical effectiveness and greater costs) [36, 44] and C2 (evidence of no difference in clinical effectiveness and greater costs) [28, 35, 47] and C3 (evidence of less effectiveness and greater costs) [24]. Acupuncture mapped in C1 [44], indicating greater clinical effectiveness with greater cost, when compared with exercise and advice. However, the same study sought to compare true and non-penetrating acupuncture and found no difference in clinical effectiveness and no difference in costs (mapping in B2). Mud-based therapy for pain management in knee OA [46] mapped in A1, reflecting the research findings that clinical effectiveness of standardized care was enhanced by the addition of mud-based therapy to standardized care.

**Table 4:** Quality of economic evaluation and clinical reporting in the studies included in the review

	Quality score		
Citation	Economic (%)	Clinical (included) (%)	Clinical (separate article) (%)
Barton *et al.* (2009) [23]	85^a^		14^c^
Bennell *et al.* (2016) [24]	100^a^	86^a^	
Ciani *et al.* (2017) [46]	92^a^		71^a^
Cochrane *et al.* (2005) [25]	83^a^	71^a^	
Coupé *et al.* (2007) [26]	92^a^		86^a^
Hurley *et al.* (2007) [28, 53]	100^a^		86^a^
Hurley *et al.* (2012) [27]	100^a^	86^a^	
Jessep *et al.* (2009) [29]	55^b^	57^b^	
Juhakoski *et al.* (2011) [30]	46^c^	71^a^	
Lord *et al.* (1999) [31]	100^a^		14^c^
Losina *et al.* (2015) [32]	87^a^		71^a^
Marra *et al.* (2014) [33]	100^a^		57^b^
Mazzuca *et al.* (1999) [34]	67^b^		57^b^
McCarthy *et al.* (2004) [21]	100^a^	71^a^	
Patel *et al.* (2009) [35]	100^a^		86^a^
Pinto *et al.* (2013) [36]	100^a^		86^a^
Reinhold *et al.* (2008) [37]	77^a^	43^c^	
Richardson *et al.* (2006)^d^ [22]	–	–	
Rome *et al.* (2004) [38]	83^a^	57^b^	
Sevick *et al.* (2000) [39]	67^b^		71^a^
Sevick *et al.* (2009) [47]	85^a^		86^a^
Stan *et al.* (2015) [40]	31^c^	0^c^	
Tan *et al.* (2016) [41]	100^a^	57^b^	
Thomas *et al.* (2005) [42]	92^a^		71^a^
Torkki *et al.* (2002) [43]	50^b^	71^a^	
Whitehurst *et al.* (2011) [44]	100^a^		71^a^
Witt *et al.* (2006) [45, 62]	31^c^		57^b^

a Quality score as a percentage of eligible items: 70–100%

b 50–70%

c <50%

d see McCarthy, 2004.

**Table 5:** Matrix of reported clinical and cost effectiveness evidence

			Declining effectiveness		
			**→**	**→**	**→**
			**1 (evidence of greater effectiveness)**	**2 (evidence of no difference in effectiveness)**	**3 (evidence of less effectiveness)**
Increased cost	↓	A (evidence of cost savings)	[21] Water-based exercise *vs* usual care for hip OA and knee OA [24] Exercise-based rehabilitation programme *vs* usual care for knee pain [38] Exercise and telephone support *vs* telephone support for knee pain [42] Mud-bath therapy added to standard care *vs* standard therapy alone		
	↓	B (evidence of no difference in costs)	[24] Exercise-based rehabilitation programme *vs* usual care for knee pain [20] Pain coping skills training/exercise *vs* exercise for knee OA [20] Pain coping skills training/exercise *vs* PCST for knee OA	[22] Behavioural graded activity *vs* usual care for hip OA and knee OA [17, 18] Class-based exercise programme + home exercise programme *vs* home exercise programme for knee OA [40] Advice and exercise plus true acupuncture *vs* advice and exercise plus non-penetrating acupuncture for knee OA	
	↓	C(evidence of greater costs)	[32] Manual therapy *vs* usual care for hip OA and knee OA [32] Exercise therapy *vs* usual care for hip OA and knee OA [32] Manual and exercise therapy *vs* usual care for hip OA and knee OA [43] Diet and exercise *vs* healthy lifestyle control [40] Advice and exercise plus true acupuncture *vs* advice and exercise for knee OA	[24] Group-based exercise-based rehabilitation programme *vs* individual-based exercise programme for knee pain [31] Arthritis self-management programme plus an education booklet *vs* education booklet (reflects standard care) for hip OA and knee OA [62] Diet *vs* healthy lifestyle control for knee OA [62] Exercise *vs* healthy lifestyle control for knee OA	[20] Pain coping skills training *vs* exercise for knee OA
	↓	D (not enough evidence on costs)			

Matrix adapted with permission from the hardcopy of Donaldson, C., Mugford M. & Vale L., Evidence-based health economics: from effectiveness to efficiency in systematic review. 1st edn. 2002: BMJ Books. 168 (8), now available as an eBook from Wiley. Evidence of clinical and cost effectiveness reported in studies included in the review with appraised quality score between 70 and 100%.

## Discussion

The findings of this review provide an overview of the characteristics and reporting quality of economic evaluation of conservative interventions for common lower extremity MSK conditions. Twenty-six unique studies that assessed the clinical effectiveness and cost effectiveness of conservative, non-pharmacological and non-surgical rehabilitative interventions for lower limb MSK conditions were identified and appraised. Despite a deliberately broad-scope search strategy, it is of note that the overwhelming majority of studies evaluated treatments for hip OA and knee OA involving an exercise component, with only two being focused on common disorders of the foot and ankle, and one on chronic pain (with OA of the hip or knee included in the range of conditions). This is unsurprising given the prevalence of hip/knee OA problems in populations [65] and the medical priority to slow disease progression towards knee and hip replacements at end-stage disease.

The reporting quality for economic evaluation was generally in accordance with clinical reporting quality recommendations, whether published as a separate economic evaluation article or within the clinical article, with a few exceptions. Of those that were judged to be less well reported for economic evaluation than for clinical effectiveness, this might have been a consequence of the scale of the research programme and study objectives. Pilot and feasibility studies are typically conducted with smaller samples, and objectives are inherently different from those of definitive randomized controlled trials. Others faced restrictions on the type and scope of economic analysis that can be conducted when data have not been collected explicitly for economic evaluation as part of the original study design. This was the case for Juhakoski *et al.* [30], who conducted a *post hoc* economic evaluation using information collected during the clinical study. In addition, consideration should be given to whether weaker reporting quality might also be, in part, a consequence of translation (for example, when an article has a dual language abstract [40]).

To make decisions about resources, it is useful to present information on costs and outcomes for each of individual intervention arm with the comparator. For example, Barton *et al*. [23] involved multiple trial intervention arms: usual care provision compared with dietary intervention, with strengthening exercises, and with a combination of diet and exercise. The use of a comparator that is equivalent to standard (or usual) care provides a pragmatic result that can be used for making policy decisions about resource allocation. However, when studies involve more than one intervention and a comparator that is not usual care, the external validity of both the randomized controlled trial and the economic evaluation become limited.

The outcome measures adopted by included studies were largely appropriate for the evaluations of interventions’ clinical and cost effectiveness. Provided that sufficient validation and evaluation of measurement properties have been undertaken, patient-reported outcome measures provide a means by which to assess and quantify the health consequences of health care for patients with specific conditions. In contrast, economic evaluation requires comparability across different disease conditions via use of a common metric. Quality-adjusted life years provide a common metric and can be calculated using a preference-based single index measure for health. These can be collected using generic preference-based measures, such as EQ-5D (used by the majority of included studies) and also by conversion to SF-6D from SF-36 and SF-12. However, the measurement properties of the generic preference-based measure (i.e. EQ-5D) for specific conditions should be known/evaluated before use in that specific clinical context. The lack of specificity of generic preference-based measures has been highlighted as a concern [66]. Given the potentially small and subtle changes that occur after conservative interventions for MSK conditions, accurate estimation of improvements is important to estimate both the burden and the consequent impact of health-care treatments. The possibility of ceiling effects limiting sensitivity to small changes in health has led to the development of a new five-level version of EQ-5D, EQ-5D-5L. This may prove more useful for health outcomes research in an MSK population in future owing to the ability to discriminate better between full health states, particularly for domains such as mobility [67]. Research to understand the full implication of using EQ-5D-5L and its value sets for quality-adjusted life year calculations is supported by the National Institute for Health and Care Excellence (NICE) [68].

The paucity of evidence about cost effectiveness of conservative, non-pharmacological and non-surgical rehabilitative interventions for the range of lower limb MSK conditions is a concern. Consistent pressures on demand for health care worldwide, coupled with a changing landscape owing to demographic and health-care developments, make the need for evidence concerning clinical and cost effectiveness more pertinent. Including economic evaluation in clinical trial design will build the evidence base about clinical and cost effectiveness. Presenting the evidence in a form such as the matrix used for this review aids decision-makers in considering clinical and economic evidence together. The ideal intervention would be in A1, where it would be both more effective and use fewer resources, but C1 is typically where new treatments map. Often, a new intervention offers improvements in outcomes but generally will also cost more (i.e. increased resource use). The studies included in this review mainly fall into C1 and C2. Presentation of information about clinical and cost effectiveness in a matrix is intended to facilitate discussions about ways to achieve maximal health gain through resource allocation decisions. C1 indicates greater costs with greater effectiveness. To make use of this, decision-makers should also consider specific health-care system implications (for costs) at their local system level, and country-specific cost-effectiveness thresholds (explicit or implicit). C2 costs more and does not deliver outcomes any better than the comparator (in the trial); therefore, it would be advisable not to introduce this intervention.

We intended this systematic review to be broad in scope, to encompass any type of economic evaluation of any conservative intervention for any lower extremity MSK condition of mechanical aetiology. To focus on a specific disease and a specific physical location using a PICO-type (Patient, Intervention, Comparator, Outcome) strategy would have narrowed the returned titles but at the cost of restricting confidence that all relevant studies had been identified.

There are limitations to this systematic review that are worth highlighting. Restricting studies to conservative treatments excluded co-provision of treatments (e.g. exercise therapy with pharmacological treatment). This was purposeful in order to determine the reported clinical effectiveness of conservative treatment. It might be that co-provision of treatment would be more aligned to real-world health-care practice and should be considered. The desire to consider clinical effectiveness meant excluding economic evaluations of interventions undertaken in general populations. Research of this nature is often focused on preventative measures, and the economic interest is prediction of prevented demand and avoided costs, rather than management of existing health-care budgets given current demand for health care.

The dominance of exercise-based interventions for MSK conditions of the hip and knee, with few economic evaluations of other clinically important lower extremity conditions, such as foot and ankle disorders, highlights a gap in the literature and therefore current knowledge. Common MSK conditions of the lower leg, such as Achilles tendinopathy or plantar fasciitis, are prevalent [69–71] and have resource implications for health-care systems. It would appear that the body of clinical evidence for conservative interventions for conditions such as these [72, 73] is not currently complemented by economic evidence, although the reasons for this are unclear.

## Supplementary Material

Supplementary Data
