# Supplementary material for: A systematic review of economic evaluations of conservative treatments for chronic lower extremity musculoskeletal complaints
Source: Rheumatol Adv Pract. 2018 Sep 10;2(2):rky030. doi: 10.1093/rap/rky030 (PMC6649923; doi:10.1093/rap/rky030)
Supplement: Supplementary Data [file rky030_supp.docx]

# SUPPLEMENTARY DATA

# Supplementary table S1. Systematic review search terms

|  | intervention OR trial OR study |
| --- | --- |
| AND | economic OR economics OR cost OR costs OR cost-effectiveness OR CEA OR CER OR benefit OR benefits OR consequences OR quality of life OR QALY OR QALYs OR HRQOL OR life quality OR life year OR life years |
| AND | lower limb OR lower limbs OR lower extremity OR foot OR feet OR foot joint OR foot joints OR knee OR knees OR leg OR legs OR ankle OR ankles OR ankle joint OR rearfoot OR hindfoot OR midfoot OR forefoot OR forefeet OR toe OR toes OR hip OR hips OR thigh OR thighs OR heel or heels OR shin OR shins OR tendon OR tendons OR ligament OR ligaments OR bursa OR meniscus |
| AND | musculoskeletal OR deformity OR deformities OR pain OR stiffness OR muscle weakness OR inflammation OR inflammatory OR fatigue OR arthritis OR osteoarthritis OR tendinopathy OR tendinosis OR tendinitis OR fasciitis OR fasciopathy OR enthesitis OR enthesopathy OR bursitis OR metatarsalgia OR neuroma OR neuritis OR posterior tibial tendon dysfunction OR tibialis posterior dysfunction OR haglund* OR osteochondritis OR stress fracture OR insufficiency fracture |
|  |  |
| NOT | TI guidelines OR systematic review OR report OR case study |
| NOT | TI fusion OR surgery OR surgical OR replacement OR transplant OR transplantation OR graft OR screw OR amputation OR reconstruction OR operation OR angioplasty OR *ectomy OR injection OR biopsy OR arthroplasty |
| NOT | TI upper limb OR lumbar OR upper extremity OR head OR neck OR shoulder OR arm OR arms OR forearm OR wrist OR axilla OR elbow OR spine OR spinal OR hand OR finger OR chest |
| NOT | TI cancer OR vascular OR Peripheral Arterial OR arterial occlusion OR peripheral vascular OR vasculitis OR venous OR vein thrombosis OR intermittent claudication OR Parkinson* OR multiple sclerosis OR stroke OR cerebral palsy OR neuropathy OR Charcot* OR spina bifida OR ankylosing spondylitis OR rheumatoid OR Juvenile Arthritis OR scleroderma OR systemic sclerosis OR psoriatic arthritis OR gout OR fibromyalgia OR sjogren* OR osteoporosis OR diabetes OR diabetic |
| NOT | SU child OR children OR youth OR adolescent OR infant |
